# Loading and Release of Phenolic Compounds Present in Mexican Oregano (*Lippia graveolens*) in Different Chitosan Bio-Polymeric Cationic Matrixes

**DOI:** 10.3390/polym14173609

**Published:** 2022-09-01

**Authors:** Melissa Garcia-Carrasco, Lorenzo A. Picos-Corrales, Erick P. Gutiérrez-Grijalva, Miguel A. Angulo-Escalante, Angel Licea-Claverie, J. Basilio Heredia

**Affiliations:** 1 Nutraceuticals and Functional Foods Laboratory, Centro de Investigación en Alimentación y Desarrollo, A.C., Carretera a Eldorado Km. 5.5, Col. Campo El Diez, Culiacán 80110, Sinaloa, Mexico; 2 Facultad de Ingeniería Culiacán, Universidad Autónoma de Sinaloa, Ciudad Universitaria, Culiacán 80013, Sinaloa, Mexico; 3 Cátedras CONACYT-Centro de Investigación en Alimentación y Desarrollo, A.C., Carretera a Eldorado Km. 5.5, Col. Campo El Diez, Culiacán 80110, Sinaloa, Mexico; 4 Centro de Graduados e Investigación en Química, Tecnológico Nacional de Mexico/Instituto Tecnológico de Tijuana, A.P. 1166, Tijuana 22000, Baja California, Mexico

**Keywords:** chitosan, polyethylene glycol methacrylate, oregano, phenolic compounds, nanoencapsulation, bioaccessibility

## Abstract

Mexican oregano (*Lippia graveolens*) polyphenols have antioxidant and anti-inflammatory potential, but low bioaccessibility. Therefore, in the present work the micro/nano-encapsulation of these compounds in two different matrixes of chitosan (CS) and chitosan-*b*-poly(PEGMA_2000_) (CS-*b*-PPEGMA) is described and assessed. The particle sizes of matrixes of CS (~955 nm) and CS-*b*-PPEGMA (~190 nm) increased by 10% and 50%, respectively, when the phenolic compounds were encapsulated, yielding loading efficiencies (LE) between 90–99% and 50–60%, correspondingly. The release profiles in simulated fluids revealed a better control of host–guest interactions by using the CS-*b*-PPEGMA matrix, reaching phenolic compounds release of 80% after 24 h, while single CS retained the guest compounds. The total reducing capacity (TRC) and Trolox equivalent antioxidant capacity (TEAC) of the phenolic compounds (PPHs) are protected and increased (more than five times) when they are encapsulated. Thus, this investigation provides a standard encapsulation strategy and relevant results regarding nutraceuticals stabilization and their improved bioaccessibility.

## 1. Introduction

The secondary metabolites present in plants, such as phenolic compounds, better known as phytochemicals, have gained great interest in recent years since they play an important role in the prevention of different diseases such as cancer, diabetes, and obesity, which are related to oxidative stress [1]. Mexican oregano (*L. graveolens*) is an endemic species from northwestern Mexico mostly known for its culinary uses but is also a rich source of phenolic compounds that can bring great benefits to human health due to their pharmacological properties, which include the anti-inflammatory, antifungal, and antibacterial activities, among others [2,3,4]. In this subject, essential oils and polyphenols are the major secondary metabolites found in oregano that are responsible for its biological properties [2,5]. Previous works have shown that oregano polyphenols have low bioaccessibility, and in-vitro digestion assays have demonstrated that phenolic acids, flavones, and flavanones of oregano seem to be susceptible to the pH changes during each digestion stage [6,7,8]. Thus, despite the bioactive properties of oregano polyphenols, they may be affected during their journey through the gastrointestinal (GI) tract, mainly due to pH changes favoring the ionization of these compounds or they are degraded, and thus the active compounds could lose their properties [1,9,10].

The low bioaccessibility of polyphenols is a subject highly reported; hence, different alternatives have been proposed to protect these metabolites from degradation. Among the technologies useful for this purpose are: (1) spray drying (for microencapsulation), (2) biopolymeric-type systems such as pectin, alginate, gums, and chitosan used to encapsulate different phytochemical compounds such as curcumin, thymol, and carvacrol [11,12,13,14,15,16,17]. Although most of these loading and release studies have been carried out with isolated compounds, it has also been found that when there is a combination of phenolic compounds, they can create synergism between them and enhance their activity [18].

The biopolymer chitosan (CS), which is obtained from a natural polysaccharide called chitin, represents a biocompatible and biodegradable platform with outstanding performance in sorption-oriented processes. This is one of the main polymeric matrixes used for the encapsulation of synthetic or natural agents due to polyelectrolyte character, which means that the charge of its functional groups can be modified depending on the pH of the medium, where the amino and the hydroxyl groups give this character [19,20,21,22]. However, this biopolymer is insoluble in normal deionized water, limiting biological applications; regarding this, some studies have been focused on modifying these molecules with different polymers to improve their solubility, such as polyethylene glycol (PEG). PEG has been one of the polymers with better biocompatibility [23]. Modified biopolymers have shown the capacity to adhere to peptide sequences [24], growth factors [25], and the ability to control mechanical properties regardless of polymerization conditions [26].

As it is well known, PEG is one of the few polymers approved by the U.S. FDA; moreover, the polyethylene glycol methyl methacrylate (PEGMA) properties are attributed to PEG due to their similar structures [27]. In addition, in case a certain percentage passes into the blood system, these particles avoid a response by the immune system, thus expanding the areas of application [28,29]. In some approaches, the use of PEGMA is related to the increase in the lower critical solution temperature (LCST) of the synthesized copolymers, increasing the LCST at temperatures greater than 32 °C, which ensures that the matrix can remain stable at temperatures higher than the LCST in aqueous medium [30].

Based on the abovementioned, in the present work the encapsulation of phenolic compounds extracted from the aerial part of Mexican oregano (*L. graveolens*) was carried out in systems based on chitosan (CS) and chitosan modified with PEGMA (Chitosan-*block*-poly(PEGMA_2000_) (CS-*b*-PPEGMA), their release profiles at different pH levels were studied and their antioxidant activity before and after encapsulation was assessed. For that, a simple encapsulation method involving mechanical stirring was used.

## 2. Materials and Methods

### 2.1. Reagents

Polyethylene glycol methyl methacrylate 2000 g mol^−1^ (PEGMA), ammonium persulfate (APS, 98%, Sigma Aldrich, Toluca, Mexico), sodium chloride (NaCl, Jalmek, San Nicolás de los Garza, Mexico), chitosan (Low weight, 98%, Sigma Aldrich), glacial acetic acid (99.7%, Fermont, Monterrey, Mexico), deuterium chloride/deuterium oxide (D_2_O/DCl 35% by weight, 99.9% deuterium, Sigma Aldrich), Folin-Ciocalteu reagent, aluminum chloride, potassium acetate, quercetin, DPPH radical, ABTS radical, potassium persulfate, HPLC grade water, and formic acid were purchased from Sigma-Aldrich (Toluca, Mexico). Moreover, sodium hydroxide (97.8%, Chemical Products of Monterrey SA de CV, Monterrey, Mexico) was purchased through a local provider.

### 2.2. Chitosan Purification

CS was dissolved in an aqueous acetic acid solution at 1% in volume, up to a concentration of 10 mg mL^−1^; afterward, the mixture was filtered under reduced pressure with Büchner. CS was precipitated from the acidic solution using 1 M sodium hydroxide solution. The alkaline CS suspension was filtered under reduced pressure with a 5 µm particle cutoff filter. The CS was washed with deionized water until neutralization, frozen, and finally lyophilized (Labconco, FreeZone^®^ 1 L, Kansas City, MI, USA).

### 2.3. Plant Material and Extraction of Free Polyphenols

**Figure 1:**
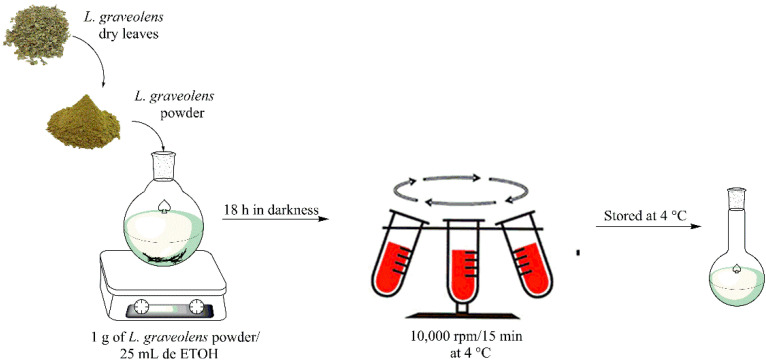
Schematic representation for free phenolic compounds extraction.

Oregano (*L. graveolens*) was collected in Santa Gertrudis, Durango, Mexico. Dried oregano leaves were ground using an Ika Werke M20 grinder (IKA, Staufen, Germany) until a fine powder consistency was obtained. Oregano powder was stored at −4 °C until use. The extract of phenolic compounds was obtained using 25 mL of absolute ethanol for 1 g of oregano powder, where the mixture was stirred and homogenized on a stirring plate (Thermo Scientific Cimarec, Waltham, MA, USA) at room temperature for 18 h. Subsequently, the extract obtained was centrifuged at 10,000 rpm for 15 min, and the supernatant was collected and stored at 4 °C until use. This technique was performed repeatedly to obtain approximately 1 L of extract. Figure 1 is a schematic representation for the process involving the extraction of phenolic compounds.

### 2.4. Characterization of the Extract of L. graveolens

#### 2.4.1. Total Reducing Capacity

The total reducing capacity was evaluated through phenolic content analysis using the Folin–Ciocalteu (FC) method proposed by Swain and Hillis [31], with some modifications. The procedure consisted of mixing 10 μL of the samples, 230 μL of distilled water, and 10 μL of FC reagent in a 96-well microplate. The mixture was incubated for 3 min, and then 25 μL of 4N Na_2_CO_3_ were added, incubating again at room temperature for 2 h in the darkness. After incubation, absorbance at 725 nm was measured (Synergy HT microplate reader). Calculations were made using a gallic acid standard curve (from 0 to 0.4 mg mL^−1^) and the results were expressed in milligrams of gallic acid equivalents per gram of powder obtained (mg AG g^−1^). Each sample was measured in triplicate (n = 3).

#### 2.4.2. Total Flavonoids Content (TFC)

The total flavonoid content was performed according to the methodology described by Ghasemi, et al. [32], with slight modifications. The process consists of taking 30 µL of the extract, then 250 µL of distilled water are added, and then 10 µL of aluminum chloride and 10 µL of 1 M potassium acetate, and it is left incubating in the darkness for 30 min; after incubation, absorbance is read at 415 nm in a Synergy HT microplate reader (Synergy HT, Bio-Tek Instruments, Inc., Winooski, VT, USA). The content of total flavonoids is determined from a quercetin standard curve (from 0 to 0.4 mg mL^−1^); the results are expressed in equivalent mg of quercetin per gram of dry extract (mg QE g^−1^ of dry sample). Each sample was measured in triplicate (n = 3).

#### 2.4.3. Antioxidant Capacity Methods

Inhibition of the 2,2-Diphenyl-1-Picrylhydrazyl Radical (DPPH)

This method uses the DPPH radical, which reduces its purple chromogen by the action of an antioxidant compound to hydrazine, a compound that colors a pale-yellow tone. This DPPH radical scavenging assay was carried out according to Karadag, et al. [33], for which 20 μL of the sample was placed in a 96-well flat-bottomed transparent microplate. Then, 280 μL of the DPPH radical were added and incubated for 30 min in the absence of white light. Finally, the absorbance at 515 nm was measured (Synergy HT microplate reader). A Trolox curve from 0.1 to 1 mmol TE g^−1^ was used to calculate the results, which are expressed as mmol Trolox equivalent per gram of powder (mmol TE g^−1^). Each sample was measured in triplicate (n = 3).

Trolox Equivalent Antioxidant Capacity (TEAC)

The antioxidant capacity by the TEAC assay of the encapsulated sample was determined as described by Thaipong, et al. [34]. ABTS was dissolved in distilled water at a concentration of 7.4 mM (stock solution). The ABTS•+ radical was produced by mixing the ABTS stock solution with 2.6 mM potassium persulfate (1:1 *v*/*v*) and incubating the mixture in the dark at 25 °C for 12–16 h before use. Subsequently, the reaction solution was prepared by taking 100 μL of the radical and dissolving in 2900 μL of solvent to adjust the absorbance. For the assay, aliquots of 15 μL of extract and 285 μL of the reaction solution were added and homogenized using a vortex. Subsequently, it was incubated in the darkness for 2 h. After the time elapsed, the absorbance at 734 nm was read in a Synergy HT microplate using transparent 96-well flat-bottom plates. The reaction solution was taken as a blank. A Trolox curve from 0.1 to 1 mmol TE g^−1^ was used to calculate the results, which are expressed as mmol Trolox equivalent per gram of powder (mmol TE g^−1^). Each sample was measured in triplicate (n = 3).

### 2.5. Identification of Phenolic Compounds by Ultra High-Resolution Liquid Chromatography/Mass Spectrometry (UPLC/MS)

Mass-liquid chromatography was used to carry out the separation for the identification of individual phenolic compounds from unencapsulated oregano extract. The analysis was performed in a class H UPLC unit (Waters Corporation, Milford, MA, USA) coupled to a G2-XS QT of mass analyzer (quadrupole and time of flight). The separation of phenolic acids was performed with a UPLC BEH C18 column (1.7 μm × 2.1 mm × 100 mm) at 40 °C, with gradient elution solution A (water-0.1% formic acid) and solution B (methanol), which is supplied at a flow rate of 0.3 mL min^−1^. On the other hand, the separation of flavonoids was performed with a different set of conditions, including a UPLC BEH C18 column (1.7 μm × 2.1 mm × 100 mm) at 30 °C, with gradient elution solution A (water-0.05% formic acid) and solution B (acetonitrile), which is supplied at a flow rate of 0.3 mL min^−1^. The ionization of the compounds was performed by electrospray (ESI), and the parameters used consisted of a capillary voltage of 1.5 kV, sampling cone: 30 V, desolvation gas of 800 (L h^−1^), and a temperature of 500 °C. A collision ramp of 0–30 V was used. The Massbank of North America (MoNA) database was used for compound identification. The identification of phenolic compounds by UPLC was performed in duplicate (n = 2).

### 2.6. Synthesis of Chitosan-Block-Poly(PEGMA)

The methodology for the synthesis of CS-*b*-PPEGMA blocks was carried out as published by Ganji and Abdekhodaie [35], with slight modifications. Briefly, the preparation was done using conventional free radical polymerization with a weight ratio of 50:50 CS:PEGMA and 0.01 M free radical initiator (KPS). CS (0.5 g), PEGMA (0.46 mL, 0.5 g), KPS (0.135 g, 0.01 M), an inert atmosphere (N_2_) in a three-necked flask with a magnetic stir bar, and 50 mL of water containing 1% (*v*/*v*) acetic acid were used. First, CS and initiator (KPS) were added followed by stirring for 30 min at 60 °C using an oil bath, and then the PEGMA_2000_ was added dropwise, and the reaction was stirred (350 rpm) for 6 h. After the reaction time, the flask was removed from the oil bath and placed in a cold-water bath. For the purification of the solution, NaOH 4M was first added to the solution to precipitate the CS; next, the product was filtered and subsequently washed with acetone to remove the residual PEGMA_2000_. Finally, the sample was dialyzed for 48 h, with changes of water periodically; after that, the recovered sample was lyophilized and weighed to determine the yield of the reaction, resulting around 70%. Chitosan-*b*-PPEGMA; ^1^H-NMR (400 MHz, CDCl_3_, δ, ppm): 4.00–4.30 (HOCH_2_CH_2_OCHCH_2_OH of the polysaccharide ring of Chitosan), 3.87 (CH_2_CH_2_O of the PEG chain), 5.13 (CHNH of acetylglucosamine ring), 3.50 (CHNH_2_ of glucosamine ring), 2.29 (CH_2_ aliphatic from the CS backbone).

### 2.7. Preparation of Nanometric Polymer Aggregates

The copolymer aggregates were prepared through a direct dissolution consisting of the solubilization of the bulk copolymer CS-*b*-PPEGMA (10 mg) in distilled water (10 mL) under magnetic stirring at room temperature for 24 h. For CS, a solution of 1 wt% was prepared in 15 mL of water with 1% (*v*/*v*) of acetic acid under magnetic stirring at room temperature for 24 h.

### 2.8. Loading of Phenolics Compounds

The loading was performed based on a solvent evaporation method [36] and adapted from the methodology reported by Picos-Corrales, et al. [37]. Briefly, 10 mg of block copolymers were dissolved in 10 mL of distilled water, and 1.5 mg of phenolic compounds were dissolved in 5 mL of ethanol. The phenolic compounds (PPHs) solution was added dropwise into the polymer solution and left under magnetic stirring for 24 h. The phenolic compounds that were not loaded were removed by centrifugation for 20 min. The purified material was filtered using a disc filter with a pore size of 1 µM and then frozen and freeze-dried. The mass of phenolic compounds loaded in the CS and block copolymers was determined by preparing a 0.3 mg mL^−1^ solution in PBS pH 2, measuring the absorbance by UV analysis at a wavelength (λ_max_) of 280 nm, and then quantified by using a calibration curve of phenolic compounds in PBS pH 2. The loading efficiency of phenolic compounds (LE) and the loaded PPHs content (LC) were calculated using Equations (1) and (2).

LE(%) = (mass of PPHs in polymer/mass of PPHs in loading solution) × 100(1)

LC(%) = (mass of PPHs in polymers/mass of dry polymer) × 100(2)

### 2.9. Measurements

*Hydrogen nuclear magnetic resonance* (^1^H-NMR) spectra were collected on a Bruker AMX-400 (Bruker Corporation, Billerica, MA, USA) (400 MHz) spectrometer and reported in ppm using tetramethylsilane (TMS) as the NMR reference standard. The solvent used was deuterium chloride (37%), D_2_O + DCl, for all samples.

*Thermogravimetric analysis* (TGA) was performed on a TA-Instruments Discovery-TGA equipment (TA-Instruments, New Castle, DE, USA). Measurements were performed for cationic matrixes and phenolic compounds loaded by heating under nitrogen flow from room temperature up to 600 °C using a heating rate of 10 °C min^−1^.

*Differential Scanning Calorimetry* (DSC) was performed on a TA Instrument DSC2000, New Castle, DE, USA). Measurements were performed for cationic matrixes and were used to determine the melting point (T_m_), the glass transition temperature (T_g_) and thermal decomposition temperatures (T_d_). For measurements, samples were cooled to −10 °C; maintained isothermally for 5 min, and afterward heated with modulation (±0.5 °C every 60 s) at a rate of 5 °C min^−1^ to 375 °C in a nitrogen atmosphere using. Two cycles of measurement were run, and the results reported corresponded to the second cycle.

*Dynamic light scattering* (DLS) measurements were carried out on 1.0 mg mL^−1^ block copolymer and 1 wt% of CS solutions at 25 °C using a Malvern Instruments Nano-ZS Nanosizer (ZEN 3690) (Malvern, Worcestershire, UK) equipment. The instrument is equipped with a helium-neon laser (633 nm) with size detection between 0.6 nm and 5 μm. DLS experiments were performed at the scattering angle of 90°, and the distribution of sizes was calculated using Malvern Instruments dispersion technology software, based on CONTIN analysis and Stokes-Einstein equation for spheres as usual.

*UV–Vis spectra* of Phenolics Compounds (PPHs) dispersions for the measurement were acquired using a UV-Vis Varian Cary 100 spectrophotometer system (Agilent Technologies, Santa Clara, CA, USA) at room temperature from aqueous dispersion.

*The Zeta potential* (ζ) of PPHs and PPHs-loaded polymeric matrixes dispersions (at 1 mg mL^−1^) was measured using a Malvern ZetaSizer Nano ZS instrument (Malvern, Worcestershire, UK). The measurements were the average of three runs performed at 25 °C and distilled water.

### 2.10. In Vitro Release Studies

For release profile studies, 0.3 mg mL^−1^ of PPHs-loaded polymeric matrixes were dispersed in 10 mL of distilled water (pH ~5 for CS-*b*-PPEGMA@PPHs) or aqueous acetic acid solution (1% *v*/*v*) (pH ~4.5 for CS@PPHs) and then added to a dialysis tube (Spectra/Por^®^ MWCO: 12–14 KDa, diameter 10 mm, Spectrum, Los Angeles, CA, USA). The dialysis tube was introduced into a 100 mL release medium, with mixture of 30% ethanol and 70% PBS inside an Erlenmeyer flask. The flask was placed in a shaking bath (Shel Lab, model SWBR17, Sheldon Manufacturing, Inc., Cornelius, OR, USA), operating at 37 °C and a shaking speed of 100 rpm. Medium aliquots of 2 mL were taken out at different times and replaced by 2 mL of fresh PBS at every sampling point. The released fraction of phenolic compounds was calculated from UV measurements at λ_max_ = 280 nm and 320 nm depending on the pH of the release medium and was then quantified using a calibration curve of phenolics compound in PBS.

### 2.11. In Vitro Gastrointestinal Digestion

A simulation of gastrointestinal digestion was performed according to the static in-vitro digestion method reported by Brodkorb, et al. [38]. This standardized procedure simulates the physiological conditions in the mouth, stomach, and small intestine, mimicking the chemical and pH conditions. Briefly, 1 mg of sample was mixed with simulated salivary fluids (SSF), then pH was adjusted to pH 7 with 6M NaOH; after that, the mixture was incubated for 5 min at 37 °C. Then, 1 mL of simulated gastric fluids (SGF), pH was adjusted to pH 3 and incubated for 2 h at 37 °C. Finally, 2 mL of simulated intestinal fluids (SIF) were added, pH was adjusted to pH 7, and the mixture was incubated for 2 h at 37 °C. In the final digestion step, the samples were centrifuged (9390× *g* at 4 °C for min), and the supernatant was collected and freeze-dried. After that, samples were resuspended in ethanol for antioxidant capacity assays.

## 3. Results and Discussion

### 3.1. Characterization of the Phenolic Compounds Present in L. graveolens

The results obtained from the nutraceutical characterization of the phenolic compounds in *L. graveolens* obtained by maceration in ethanol are found in Table 1, where the extracted compounds show antioxidant capacity. It can be seen that the extracted compounds have antioxidant activity against the different free-radical DPPH, AAPH, and ABTS. Comparing our results with the literature (TRC, 51.26 ± 2.36 mg EAG g^−1^; TFC, 11.80 ± 0.12 mg QE g^−1^; DPPH, 500.54 ± 9.63 µmol TE g^−1^; ORAC, 812.31 ± 35.46 µmol TE g^−1^; and ABTS, 350.07 ± 0.45 µmol TE g^−1^) [9], it was observed that the TRC of both extracts is very similar, showing differences mainly in the TFC where a lower value was registered with the extraction in absolute ethanol. Cortes-Chitala, et al. [39], reported that the TRC of the *L. graveolens* was around 99.71 mg AGE g^−1^ dried weight; the higher reducing activity might be mainly because they used ethanol:water (58:42) as a solvent for extraction, plus the region where the sample was taken is different [39].

#### Characterization by UPLC-MS

**Figure 2:**
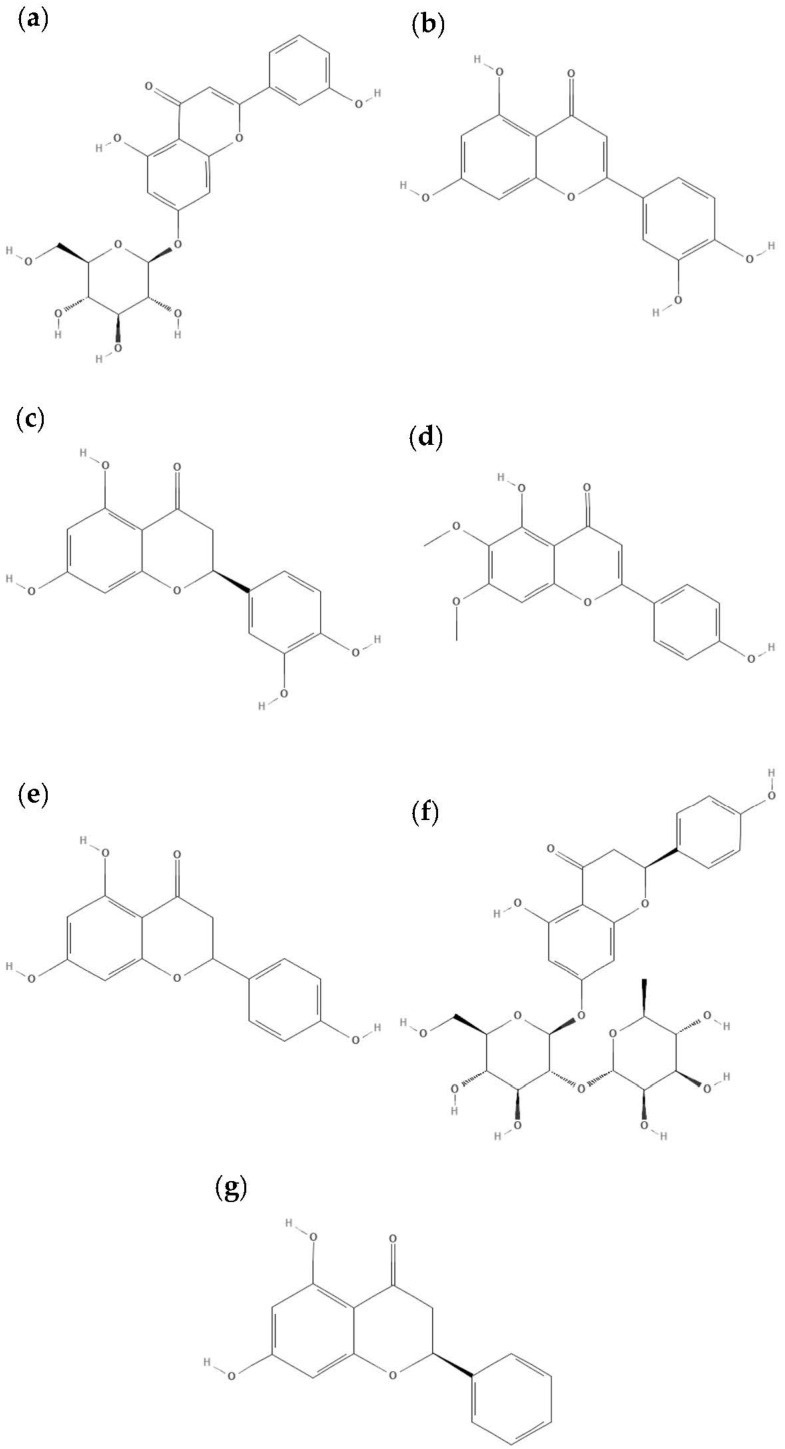
Chemical structures of some compounds contained in the phenolic extract characterized by UPLC-MS: (**a**) cosmoside, (**b**) luteolin, (**c**) eriodyctiol, (**d**) cirsimaritin, (**e**) naringenin, (**f**) naringin, (**g**) pinocembrin.

The *L. graveolens* ethanolic extract was characterized by UPLC-MS, where it was possible to observe that the sample had a diversity of flavonoids and some phenolic acids (Table 2). Some of the identified compounds are flavones such as luteolin-glycoside, cosmoside, eriodictyol, and cirsimaritin; others are flavanones such as naringin, naringenin, and pinocembrin (Figure 2). The phenolic profile in this study is consistent with previous studies in *L. graveolens* extracts [9,39,40]. However, the distribution and content of phenolic content in our study could be different, owing to factors such as recollection time and the extraction method used for phenolic recovery. Moreover, in previous studies, the most abundant phenolics in methanolic extracts were naringenin, kaempferol-glucoside, kaempferide, caffeic acid, cirsimaritin, kaempferol, and taxifolin; most of these molecules were also found in our extracts. These metabolites have been associated with anti-inflammatory, antiapoptotic, and antimicrobial activity [4,39,41].

### 3.2. Synthesis and Characterization of Cationic Matrixes

#### 3.2.1. Chitosan

**Figure 3:**
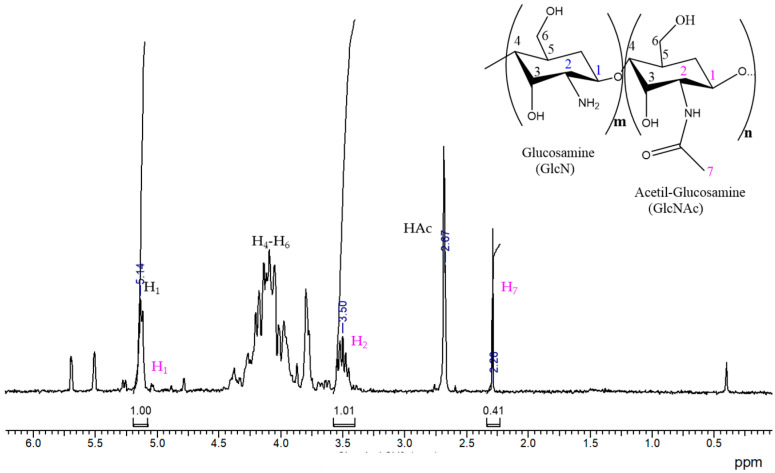
^1^H-NMR spectrum of chitosan in D_2_O/DCl at 30 °C.

After performing the purification of CS by precipitation in aqueous medium, it was characterized by ^1^H-NMR (Figure 3). The assignment of the hydrogens corresponding to CS, which is a polysaccharide containing units of glucosamine (GlcN) and acetyl-glucosamine (GlcNAc), is the following: at 2.36 ppm, the signal corresponds to the hydrogens of the methyl group of the acetyl-glucosamine units (H_7_); a signal with an intensity of around 2.67 ppm can also be observed, which corresponds to the signal of residual acetic acid (HAc), which comes from the purification of CS and was difficult to remove completely. Around 3.5 ppm, a multiplet is observed, and the signal is associated to the alpha hydrogen signal to the amino group of the repeating unit of GlcN (H_2_); the signals located between 3.55 and 4.5 ppm correspond to the hydrogens from the polysaccharide ring (H_3_-H_6_); and the signal at 5.14 ppm corresponds to the H_1_ of the repetitive unit of glucosamine [42].

The degree of deacetylation (*DD*) of CS was calculated with Equation (3), using the integrations under the curve of the signal of H_1_ (GlcN) and H_7_ (GlcNAc), which gave us an 88% deacetylation degree of CS.

After obtaining the intrinsic viscosity, the viscosity-average molecular weight was calculated using the Mark–Houwink–Sakurada constants, with *K* and *a* being 2.14 × 10^−3^ mL g^−1^ and 0.657, respectively [43]. As a result, the viscosimetric molecular weight obtained was 114 KDa, classified as relatively low molecular weight, which is suitable for using CS as a nanocarrier of different drugs.

(3) $$DD=\left\{\left(\frac{H_{1\left(GlcN\right)}}{H_{1\left(GlcN\right)}+\frac{H_{7\left(GlcNAc\right)}}{3}}\right)\times 100\right\}$$

#### 3.2.2. Chitosan-b-PPEGMA

**Figure 4:**
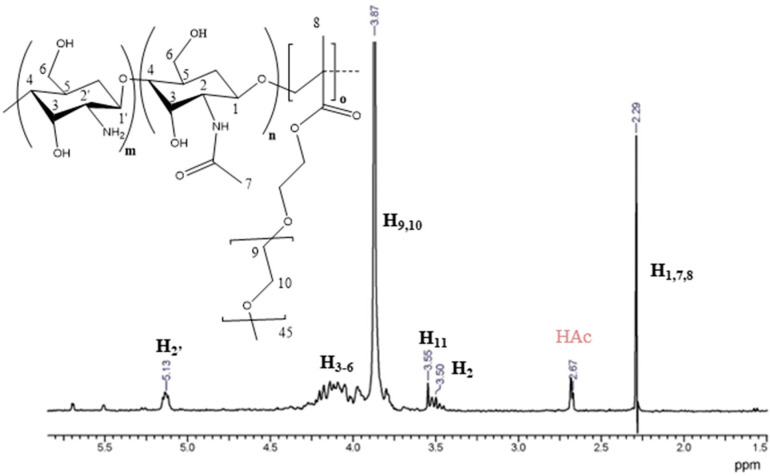
^1^H-NMR spectrum of chitosan-*b*-poly(PEGMA_2000_) in D_2_O/DCl at 30 °C.

The block copolymer based on CS and PEGMA_2000_, was prepared following the methodology reported by Ganji and Abdekhodaie [35] applying some modifications. The product was characterized by ^1^H-NMR (Figure 4). In the spectrum, the intense signal can be highlighted at 3.87 ppm that corresponds to the hydrogens (H_9_ and H_10_) of the repetitive ethoxy units in PEGMA_2000_; and the signal of lower intensity at 3.55 ppm (H_11_) corresponds to the hydrogen of methylene bound to oxygen at the end of the PPEGMA chain. Besides, the signals previously described for the chitosan backbone are also observed. This analysis indicated that the synthesis of chitosan-*b*-poly(PEGMA_2000_) was successfully accomplished.

The percentage of PEGMA_2000_ present as polyPEGMA_2000_ block was determined by ^1^H-NMR. For that, the integration from 3.6 ppm to 4.4 ppm was related to the signal at 5.15 ppm, which turned out to be higher than 90%, which makes the copolymer very soluble in an aqueous medium. This is an important factor when using this type of biopolymer in different pharmacological applications because of its good reservoir capacity for loading different drugs. Furthermore, the average size of the CS backbone was shortened during the free-radical high-temperature synthesis; this was also determined by viscosimetry in an experiment without PEGMA_2000_. The results showed that the size of the polysaccharide chains decreased by more than 90%, therefore it is not surprising that the CS content in the block copolymer is much lower than expected from the synthetic recipe [44].

#### 3.2.3. Thermal Characterization

**Figure 5:**
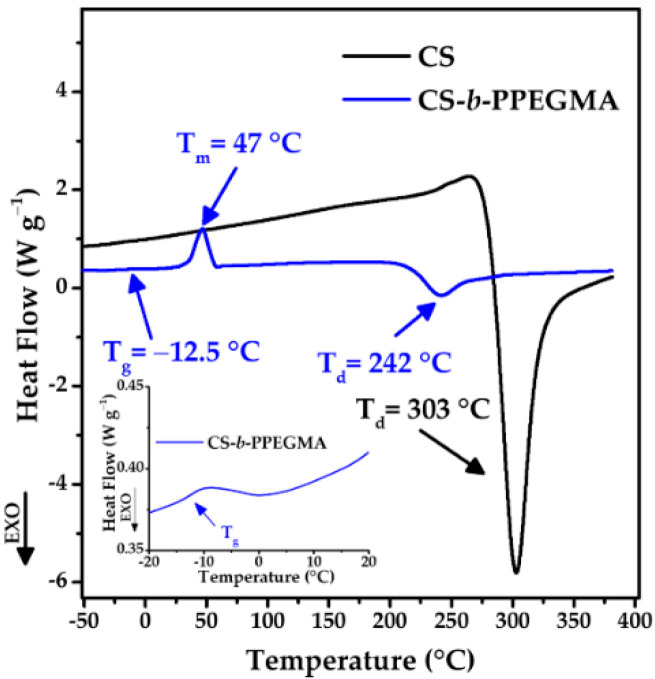
DSC thermograms of CS and CS-*b*-PPEGMA.

A change in the structure of CS can be observed in the differential scanning calorimetry analysis (Figure 5); in the respective thermogram, only one thermal event can be observed at 303 °C, which is characteristic of the decomposition of glucosamine groups [45,46]. On the other hand, in the thermogram of the CS-*b*-PPEGMA blocks, two transition temperatures can be observed: the first at −12.5 °C, representing the glass transition temperature (T_g_) of PEGMA_2000_ (an expanded view is presented), and the second at 47 °C representing the melting point (T_m_) also of PEGMA_2000_ [35,47]; a third thermal event observed at 242 °C is related to the decomposition temperature of PEGMA and CS. The decrease in the decomposition temperature of CS could be attributed to the smaller size of the CS chain after the synthetic procedure.

**Figure 6:**
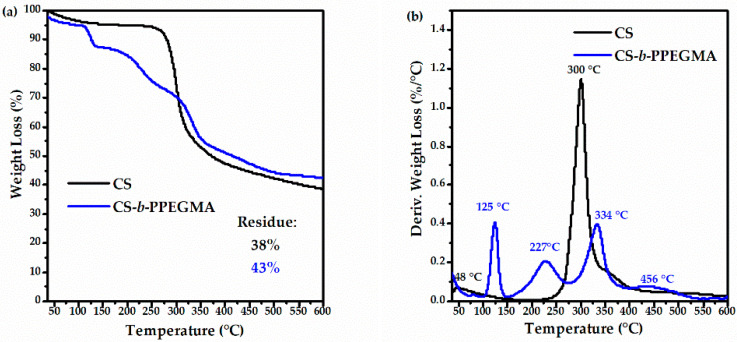
Thermograms of CS and CS-*b*-PPEGMA: (**a**) TG-thermogram and (**b**) DTG-thermogram.

Thermogravimetric analysis shows differences between both compounds, highlighting that the decomposition temperatures of CS and the CS-*b*-PPEGMA block copolymer are similar (Figure 6a). Figure 6b shows the decomposition of these biopolymers; in the case of CS, a single decomposition around 300 °C is shown [48]. As for the CS-*b*-PPEGMA block copolymer, four decomposition steps can be observed: the first one at temperatures below 100 °C, which represents the loss of moisture from the sample; at 125 °C, this first decomposition step can be attributed to the low molecular weight chains of CS that formed after breaking the backbone, the next step at 227 °C corresponds to the first decomposition of PEGMA_2000_, the third step at 334 °C corresponds to the decomposition of CS chains, and finally a weight loss at 456 °C, which would correspond to the second decomposition of PEGMA_2000_ [49,50]. In both systems, a residue of around 40% is observed, being attributed to the formation of coke.

### 3.3. Loading of Phenolic Compounds into Different Cationic Polymers

**Figure 7:**
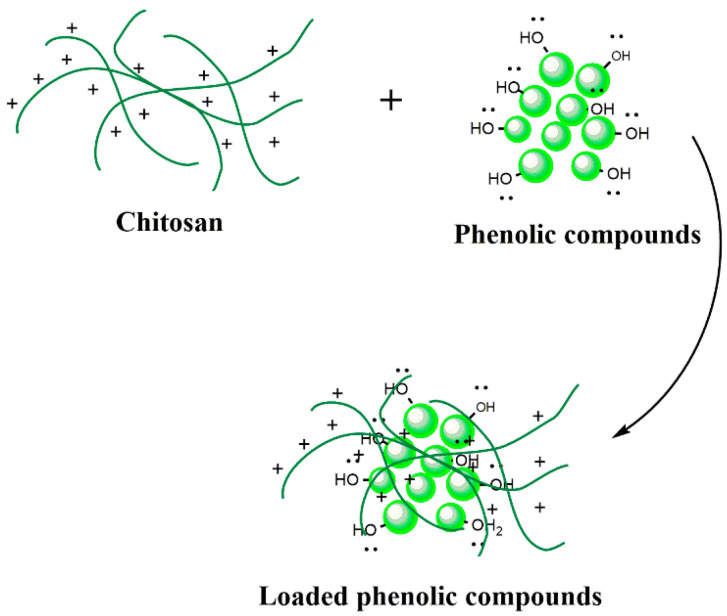
Scheme of the possible interaction between CS and the polyphenolic compounds.

After loading the different matrixes, samples were analyzed by UV-vis spectroscopy to determine the content of phenolic compounds (Table 3). It was observed that CS had a loading efficiency between 90–99%, while for the blocks of CS-*b*-PPEGMA the efficiency was lower from 50–60%; on the other hand, the loading content was 65% and 99%, respectively [51,52]. The pK_a_ of phenolic compounds varies around 6–9 [53,54,55,56,57,58], and the solubility of these is affected depending on the pH of the medium; considering that, their loading was carried out in an aqueous medium acidified with 1% v/v of acetic acid (pH = 4.5), it could be said that some of the compounds were partially soluble. An opposite case for CS with a pK_a_ around 6.17–6.51 [59,60,61], is completely soluble at acidic pH due to its protonated amines. When those amines are in contact with the hydroxyl groups (-OH) of the phenolic compounds (Figure 2) they can form hydrogen bonds or present electrostatic interactions (neutralization of charges) (Figure 7). Due to this molecular interaction in the case of CS, which has a higher content of free amines, it can encapsulate a higher concentration of the phenolic compounds; on the other hand, in the CS-*b*-PPEGMA blocks, the shorter CS chains and the fact that the ends are blocked with PPEGMA leads to less free amines per chain that can be available for interaction with the -OH of the phenolic compounds (Table 3). Moreover, a significant difference was registered in **ζ** change when a simple mixture of matrix and PPHs was prepared as compared with PPHs-loaded polymeric matrixes, so the stabilization and loading with the different matrixes can be inferred.

**Figure 8:**
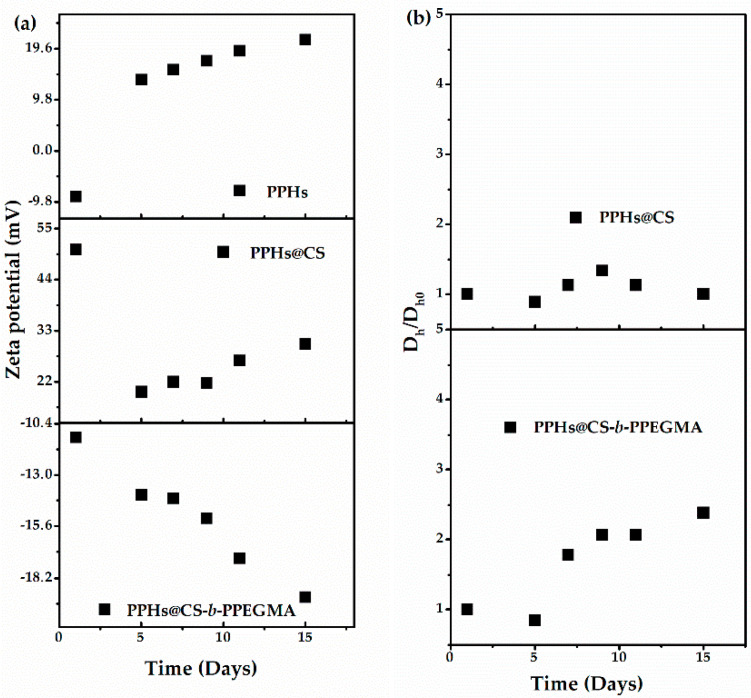
Stability study of systems loaded with phenolic compounds from *L. graveolens* zeta potential (**a**) and particle size (**b**).

To determine if these matrixes protected the phenolic compounds, the stability of these systems before and after loading was determined by zeta potential (Figure 8a) and particle size (Figure 8b) in measurements for about 15 days. From these experiments, the most stable system resulted to be the CS matrix, whilst the particle size of the CS-*b*-PPEGMA blocks increased with the time, although the latter presented good size stability for up to 5 days. On the other hand, the study of zeta potential for the two different systems and the unencapsulated polyphenols can be observed, and it was found that the zeta potential (positive or negative) of the samples increased with the time. This effect may be because most of these compounds are not soluble in aqueous medium, so they precipitate, and those partially soluble compounds can be ionized [9,12].

On the other hand, in the case of CS systems, it can be observed how there was a drastic decrease in the surface charge after day 3 (compared with day 5), which could be caused by a decrease in the number of free amino groups improving the polymer-organic compounds interactions, maintaining similar particle size and colloidal stability along the days (Figure 8). In the case of the CS-PPHs system (PPHs@CS), the variations in the zeta potential did not significantly affect the size, and formation of more complex aggregates was not detected. In general, surface charge variations depend on free groups in their ionized form or forming hydrogen bonds, belonging to the polymer or the nutraceuticals. Figure 7 shows the possible interaction between CS and the polyphenolic compounds.

#### Thermal Characterization of Phenolic Compounds

**Figure 9:**
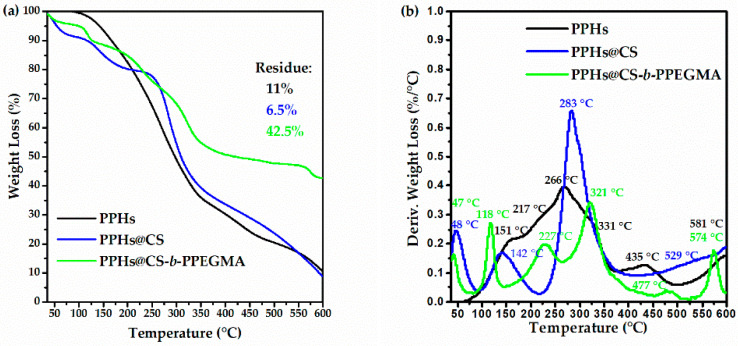
Thermal stability of loaded and unloaded PPHs: (**a**) TG-thermogram and (**b**) DTG-thermogram of Polyphenolics Compounds (PPHs), PPHs loaded in CS (CS@PPHs), and PPHs loaded in CS-*b*-PPEGMA (CS-*b*-PPEGMA@PPHs).

The stability of the PPHs in the two different cationic matrixes was determined by thermogravimetric analysis, as is shown in Figure 9. It is shown that the decomposition signals of the single PPHs sample were not overlapped with the signals of the compounds loaded in the different cationic matrixes (CS@PPHs and CS-*b*-PPEGMA@PPHs). Also, a displacement of the decomposition temperatures of the PPHs can be observed, which would be associated with the cationic matrixes protecting these systems through different interactions, which could also impart on them higher thermal stability [62].

### 3.4. Release of Phenolic Compounds

**Figure 10:**
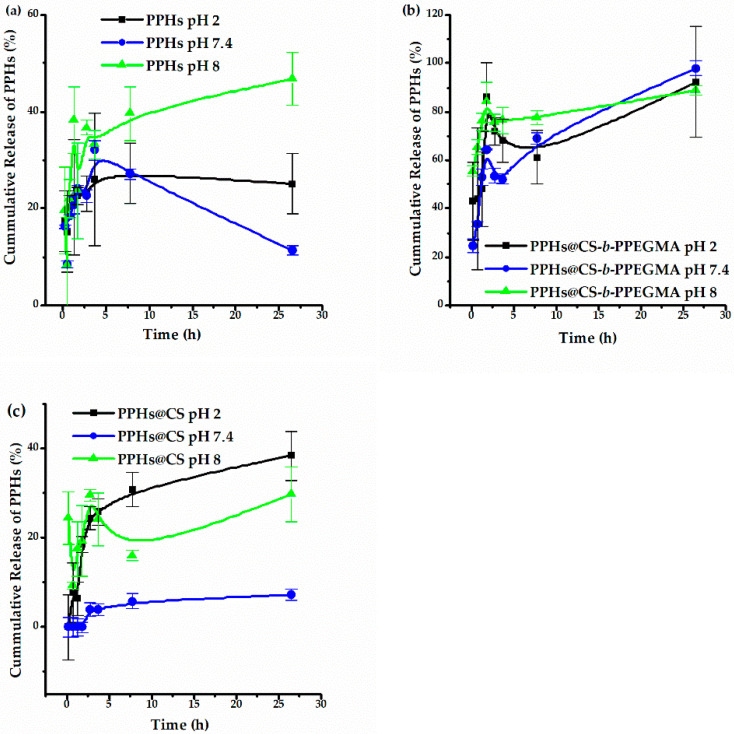
Release profiles of (**a**) PPHs, (**b**) PPHs@CS-*b*-PPEGMA, and (**c**) PPHs@CS. Values represent the mean ± standard deviation (n = 3).

The release profiles were performed by simulating pH conditions in the gastrointestinal tract using phosphate buffers with pH 7.4, 2, and 8 (Figure 10); each simulation was incubated for 24 h. Subsequently, a continuous release was also performed (pH 2, 6.8, 7.4 and 8); in the same way, the surface charge of both systems was determined at the different pH to study their behavior. In Figure 10a, it can be seen that the phenolic compounds show a higher percentage of release at pH 8, while at pH 7.4 a drop in their concentration is observed after 24 h of release; furthermore, the maximum release percentage of these compounds was 45%. It is relevant to specify that the rest of the PPHs precipitate as they are not completely soluble in an aqueous medium. According to the release profiles recorded from the two different matrixes, it can be seen that the CS-*b*-PPEGMA blocks provide a more controlled release of these compounds, releasing more than 80% of the PPHs after 24 h. On the other hand, CS only releases a maximum of around 40% of the guest compounds, and this is mainly due to the solubility of CS in the different pH values [63], observing that at pH 2, CS is completely soluble and releases a higher percentage of the phenolic compounds.

The release in all cases started quickly at the beginning and then a slower release of nutraceuticals compounds over time was recorded; this could result from a fraction of the molecules just being adsorbed onto the matrixes’ surface, triggering an initial burst release [64].

**Figure 11:**
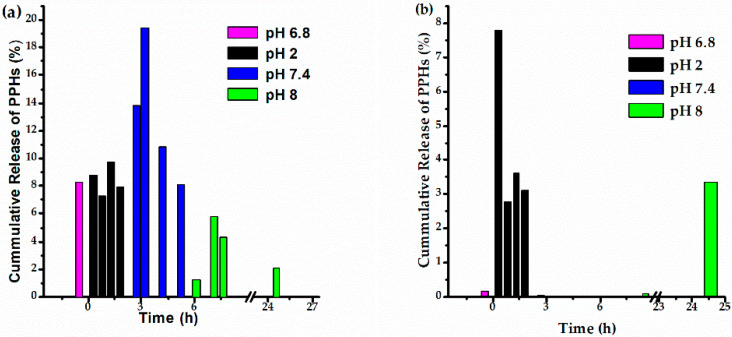
Cumulative release profiles of PPHs from CS-*b*-PPEGMA (**a**) and CS (**b**). Note: the cumulative release is only within a pH value; after every pH change, a new cumulative release is calculated.

In Figure 11, the continuous release profiles of the PPHs loaded in CS and CS-*b*-PPEGMA are shown—the systems presented different behavior when continuous release was performed, changing the pH and simulating the gastrointestinal tract passage as compared to individual pH values. It is observed that the maximum release for the CS-*b*-PPEGMA matrix was less than 20%, and in the case of CS a maximum of 8% was released; this shows a gap where more than 80% of these compounds are protected within the different matrixes [65]. The difference between the release of the different matrixes is mainly due to their solubility in the aqueous medium, as well as the solubility/stability of the phenolic compounds released in the medium; these tend to precipitate when they are in an aqueous medium. Nevertheless, there is still a possibility that the enzymes present in *in-vivo* systems can degrade these matrixes and release a higher percentage of these compounds [13].

### 3.5. In-Vitro Gastrointestinal Digestion

Encapsulation has been used both to enhance the bioaccessibility of phenolic compounds and to protect them from degradation [12]. In this sense, the properties of the charged and unloaded phenolic compounds were determined in both systems after the gastric and intestinal phase by the total reducing capacity and TEAC assays to evaluate if the compounds maintained their antioxidant properties.

Table 4 shows a summary of the results obtained, and it can be observed that both the total reducing capacity and the antioxidant capacity of the PPHs are diminished in the SGF phase. This may be mainly due to the ionization of the phenolic compounds present, which causes a decrease in their activity towards the free radical target; in the intestinal phase, a decrease is seen mainly by the ABTS assay, which may have been because a large proportion of the released phenolic compounds could have been degraded or ionized in the gastric phase [9]. During in-vitro gastrointestinal digestion, encapsulated polyphenols can be partially released from the matrix; in this case, the matrix material can be affected by the pH changes during each digestive stage, CS matrix is most affected by this change in pH since it changes its solubility as it passes through the GI tract. At low pH, it is completely soluble, while at neutral-basic pH it is the opposite. The released polyphenols from the CS matrix are exposed to the different pH in the simulated gastrointestinal solutions, undergoing deprotonation of their chemical structures and partial hydrolysis, which has also been reported. Deprotonated polyphenols can affect the way they interact with the targeted free radicals in each antioxidant assay. The rate at which polyphenols are affected by the gastrointestinal environment is dependent on many factors, and one of the most reported is the matrix in which they are contained. In this work, we used the TEAC assay, which is based on the transfer method and depends on the reducing capacity of the evaluated substance [66,67].

In this approach, the encapsulated phenolic compounds have a higher activity than when they are not protected by one of the different matrixes [68]. In the same way, little difference can be observed between both matrix systems, and this may be mainly due to the effect of the surface charge that each system presents, causing the final TCR to be higher in the CS-*b*-PPEGMA matrix, having a negative partial charge because the amines are not protonated, and a higher concentration of these compounds remains inside; nonetheless, CS has a positive surface charge due to the number of amines that can be protonated, showing lower activity. The opposite is seen in the results obtained by TEAC, but in this case, it could be because the CS has a greater amount of compounds within the matrix; namely, there is a greater PPHs fraction compared to the block copolymers [52].

Moreover, it has been reported that CS-based polymers improve the disturbance in glucose metabolism in diabetic mice, such as reducing blood glucose, reversing insulin resistance, enhancement of the colonic epithelial integrity, and a modulatory effect on the gut microbiota. In addition, CS-based polymers loaded with phenolic compounds have been proposed as promising nanochemopreventive agents against cancer. Also, CS has been reported as a permeability enhancer, which must be addressed to evaluate if phenolic-loaded CS can increase the cell permeability of phenolics. Thus, CS and CS-based polymers loaded with phenolic compounds can result in multiple beneficial effects in the development of functional foods [11,41,69,70].

## 4. Conclusions

From the characterization, it can be concluded that the synthesis of block copolymers based on CS and PEGMA was successfully accomplished. The phenolic compounds were efficiently encapsulated with the CS and CS-*b*-PPEGMA matrixes, which was verified by determining the surface charge (zeta potential) of the colloidal systems before and after the loading. This change is derived from the type of interaction taking place between the -OH groups of the phenolic compounds and the –NH_2_ onto the CS backbone in the different aqueous medium. PEGMA_2000_ led to the development of formulations having smaller particle size; however, CS plays a key role improving the matrix–guest compounds interactions and colloidal stability. The CS-*b*-PPEGMA matrix allowed a higher percentage of release, which can be attributed to the improved solubility of these platforms, as compared with single CS, and during cumulative release experiments only a maximum of 20% of active compounds were released from this platform; namely, 80% of the guest substances remained trapped in the polymeric matrix after the gastric and intestinal phases, indicating that the CS-*b*-PPEGMA may offer greater protection for the active compounds in the gastric phase. This could be related to the negative surface charge of this matrix undergoing delayed protonation, and an inverse effect was exhibited by CS. Thus, the results demonstrated that the encapsulation of nutraceuticals can improve their stability, solubility, and activity, and the CS-*b*-PPEGMA matrix can help improve the controlled release of compounds present in Mexican oregano (*Lippia graveolens*).

## Figures and Tables

**Table 1:** Nutraceutical results of the ethanolic extract of oregano (*L. graveolens*).

TRC * (mg GAE g^−1^ Dried Oregano Leaf)	TFC ** (mg QE g^−1^ Dried Oregano Leaf)	DPPH ***	ORAC ***	ABTS ***
		(µmol TE g^−1^ Dried Oregano Leaf)		
50 ± 5.5	0.59 ± 0.019	339 ± 26.56	2639 ± 12.7	476 ± 1.27

* Total Reducing Capacity (TRC), ** Total Flavonoids Content (TFC) *** DPPH and ABTS are scavenging capacity, and ORAC is antioxidant capacity. Values represent the mean ± standard deviation (n = 3).

**Table 2:** Flavonoids from the ethanolic extract of *L. graveolens* characterized by UPLC-MS.

Compound	Compound Type	Molecular Mass [M-H]^-^
Luteolin-glucoside	Flavone	447.1
Cosmoside	Flavone	431.1
Naringin	Flavanone	579.17
Quercetin	Flavonol	301.04
Kaempferol	Flavonol	285.04
Eriodictyol	Flavone	287.06
Naringenin	Flavanone	271.06
Pinocembrin	Flavanone	255.07
Taxifolin	Flavanonol	305.05
Cirsimaritin	Flavone	313.07

**Table 3:** Loading Efficiency (LE) and Loading Content (LC) of PPHs in polymer matrixes; zeta potential (ζ) and particle sizes of loaded (@PPHs) and unloaded (Matrix) polymer matrixes. Values represent the mean ± standard deviation (n = 3).

Polymer	LE (%)	LC (%)	ζ (mV)			D_h_ (nm)	
			Mixture	@PPHs	Matrix	@PPHs	Matrix
CS	90–99	65	10.2 ± 4.32	50.4 ± 3.27	55.4 ± 5.07	1106 ± 87	955 ± 75
CS-*b*-PPEGMA	50–60	99	7.91 ± 4.37	−15.5 ± 4.57	−9.07 ± 4.86	458 ± 0.01	190 ± 17
PPHs			−8.79 ± 4.29	-	-	-	-

**Table 4:** Results of the total reducing capacity (TRC) and Trolox equivalent antioxidant capacity (TEAC) of phenolic compounds loaded in chitosan (PPHs@CS), phenolic compounds loaded in CS-block-poly(PEGMA) (PPHs@CS-*b*-PPEGMA), and non-encapsulated phenolics (PPHs) after gastric and intestinal in-vitro digestion.

	TRC * (mg QE g^−1^ Dried Oregano)		TEAC ** (µmol TE g^−1^ Dried Oregano)	
	SGF	SIF	SGS	SIF
PPHs@CS	81.19 ± 4.18 ^b^	111.70 ± 9.90 ^a^	163.12 ± 79.11 ^b^	446.56 ± 9.01 ^a^
PPHs@CS-*b*-PEGMA	89.03 ± 2.39 ^b^	135.50 ± 3.15 ^a^	79.15 ± 23.15 ^b^	415.79 ± 7.07 ^a^
PPHs	16.68 ± 1.41 ^b^	50.54 ± 2.90 ^a^	104.19 ± 0.22 ^a^	100.82 ± 7.99 ^a^

* Total Reducing Capacity (TRC), ** Trolox equivalent antioxidant capacity (TEAC) is a scavenging capacity. Values represent the mean ± standard deviation (n = 3). ^a, b^ means are significantly (α > 0.05) different according to the Tukey test.

## Data Availability

The data that support the findings of this study are available from the corresponding author upon reasonable request.
